# Characterization and elimination of linkage-drag associated with Fusarium wilt race 3 resistance genes

**DOI:** 10.1007/s00122-021-03810-5

**Published:** 2021-03-30

**Authors:** Jessica Chitwood-Brown, Gary E. Vallad, Tong Geon Lee, Samuel F. Hutton

**Affiliations:** 1 Gulf Coast Research and Education Center, Institute of Food and Agricultural Sciences, University of Florida, 14625 CR 672, Wimauma, FL 33598-6101 USA; 2 Horticultural Sciences Department, University of Florida, Gainesville, FL 32611 USA; 3 Plant Molecular and Cellular Biology Graduate Program, University of Florida, Gainesville, FL 32611 USA

## Abstract

**Key message:**

Reducing the size of the I-3 introgression resulted in eliminating linkage-drag contributing to increased sensitivity to bacterial spot and reduced fruit size. The I-7 gene was determined to have no effect on bacterial spot or fruit size, and germplasm is now available with both the reduced I-3 introgression and I-7.

**Abstract:**

Tomato (*Solanum lycopersicum*) production is increasingly threatened by Fusarium wilt race 3 (*Fol3*) caused by the soilborne fungus, *Fusarium oxysporum* f. sp. *lycopersici*. Although host resistance based on the *I-3* gene is the most effective management strategy, *I-3* is associated with detrimental traits including reduced fruit size and increased bacterial spot sensitivity. Previous research demonstrated the association with bacterial spot is not due to the *I-3* gene, itself, and we hypothesize that reducing the size of the *I-3* introgression will remedy this association. Cultivars with *I-7*, an additional *Fol3* resistance gene, are available but are not widely used commercially, and it is unclear whether *I-7* also has negative horticultural associations. To characterize the effect of *I-3* on fruit size, segregating populations were developed and evaluated, revealing that the large *I-3* introgression decreased fruit size by approximately 21%. We reduced the *I-3* introgression from 5 to 140 kb through successive recombinant screening and crossing efforts. The reduced *I-3* introgression and *I-7* were then separately backcrossed into elite Florida breeding lines and evaluated for effects on bacterial spot sensitivity and fruit size across multiple seasons. The reduced *I-3* introgression resulted in significantly less bacterial spot and larger fruit size than the large introgression, and it had no effect on these horticultural characteristics compared with *Fol3* susceptibility. *I-7* was also found to have no effect on these traits compared to *Fol3* susceptibility. Together, these efforts support the development of superior *Fol3*-resistant cultivars and more durable resistance against this pathogen.

## Introduction

Fusarium wilt, caused by the soilborne fungus *Fusarium oxysporum* f. sp. *lycopersici* (*Fol*), is an important vascular disease that threatens global tomato (*Solanum lycopersicum*) production for both processing and fresh-market systems. Disease symptoms begin as pronounced chlorosis and wilting of lower basal leaves that progresses acropetally to the upper leaves, leading to a rapid decline of the plant, accelerated fruit ripening, and eventual plant death. Disease development is favored by sandy, acidic soils and warm temperatures (25–27 °C).

*Fol* produces several asexual structures, including microconidia and macroconidia, as well as thick-walled chlamydospores that can survive in the soil for up to ten years (Correll and Jones, 2014). Once introduced into a field, *Fol* is almost impossible to eliminate, as it can survive nearly indefinitely in soil as either chlamydospores in plant residues or as a common root epiphyte/saprophyte on numerous weeds without causing disease (Katan 1971). As a result, cultural control practices such as long fallow or crop rotation are largely ineffective. Chemical control via fumigation is an important management strategy to limit the impact of soilborne pathogens, including *Fol*, on tomato production (Noling and Becker 1994). From the 1970′s until 2014, the tomato industry in Florida and the Southeast relied heavily on methyl bromide for fumigation to maintain economical production levels (Ragsdale and Wheeler 1995). However, its designation as an ozone-depleting chemical in 1993 under the Montreal Protocol led to the phase out of methyl bromide usage in the US. Although several registered alternative fumigants to methyl bromide exist, all lack the broad-spectrum activity and the volatility that made methyl bromide effective, and these are also less effective for managing Fusarium wilt (Vallad et al. 2014). Host resistance is the most effective control strategy for *Fol*.

Three races of *Fol* have been described, distinguished by their response to dominant, race-specific resistance genes introgressed from wild tomato species. The *I* (for “immunity” to Fusarium wilt) and *I-2* genes, conferring resistance to *Fol* races 1 (*Fol1*) and 2 (*Fol2*), respectively, were introgressed from the wild tomato relative *Solanum pimpinellifolium*, and both are located on chromosome 11 (Bohn and Tucker 1939; Alexander 1959). Although McGrath et al. (1987) first reported resistance to *Fol* race 3 (*Fol3*) in the *Solanum pennellii* accession PI414773, Scott and Jones (Scott and Jones 1989) introgressed the *I-3* gene from the *S. pennellii* accession LA716. *I-3*, which encodes an S-receptor-like-kinase (SRLK) on chromosome 7 (Catanzariti et al. 2015), has been the primary source of *Fol3* resistance for cultivar development around the world (Lim et al. 2008). It was later determined that resistance introgressed from PI414773 is not based on *I-3* but instead on an alternative resistance gene located on chromosome 8, and this gene was designated as *I-7* (Lim et al. 2006). Notably, *I-7* encodes a leucine-rich-repeat receptor-like-protein (LRR-RLP) and is not known to recognize the same avirulence protein in the pathogen as *I-3* (Gonzalez-Cendales et al. 2016). *I-7* has not been widely used in the development of commercial cultivars, likely because *I-3* was already deployed, and it was only relatively recently that *I-7* was distinguished from *I-3*; also marker resources for selecting *I-7* were not available until recently.

Today, *I* and *I-2* are very commonly deployed, and most commercial varieties contain these resistance genes. However, resistance to *Fol3* has not been utilized as ubiquitously. *Fol3* was initially discovered in Australia in 1979 before being identified in Florida in 1982 (Scott and Jones 1989), and it now exists in tomato production regions around the world. Although cultivars containing *I-3* have been commercially available since the mid 1990′s, breeders have experienced considerable difficulty developing commercially acceptable *Fol3*-resistant hybrids. Some of the earliest *I-3* parents had greater susceptibility to blossom-end rot (Scott 2004), and smaller fruit size was demonstrated in plants that were homozygous for *I-3* compared to plants heterozygous for the gene (Scott 1999). Furthermore, Hutton et al. (2014) demonstrated that the *I-3* introgression in advanced breeding material contributed as much as 20% increased infection to bacterial spot race T4 (*Xanthomonas perforans*) in *I-3/I-3* plants relative to *i-3/i-3* plants.

Recently, Li et al. (2018) developed recombinant inbred lines (RILs) segregating for different portions of the *S. pennellii I-3* introgression from material with an introgression of more than 5 Mb in size. They found that haplotypes of RILs segregating for proximal portions of the *I-3* introgression (above the *I-3* gene) showed significantly different responses to bacterial spot, regardless of whether the *I-3* gene itself was fixed or segregating. These findings provided compelling evidence that increased sensitivity to bacterial spot is not due to the *I-3* gene itself but likely results from linkage drag.

To develop *Fol3*-resistant cultivars that meet industry standards, resistance loci that effectively control Fusarium wilt and which are free of negative associations must be made available. We hypothesize that by reducing the size of the *I-3* introgression, we will be able to remedy the association with increased sensitivity to bacterial spot, and potentially the possible negative effect on fruit size. Additionally, there is little information about the potential utility of *I-7* in commercial varieties, and it is likely that the extent to which *I-7* will be commercially adopted will depend upon whether it is free of any negative associations. The objectives for this research were to characterize the effect of the large *I-3* introgression on fruit size; reduce the *I-3* introgression and test the effect of the reduced introgression on bacterial spot sensitivity and fruit size; and also to determine whether *I-7* has any effect on bacterial spot sensitivity and fruit size.

## Materials and methods

### Field trial methodology

All field trials were conducted at the Gulf Coast Research and Education Center in Balm, FL. For each of these trials, seed was sown directly into peat-lite soilless media (Speedling, Sun City, FL) in 128-cell Proptek vegetable propagation trays (39 cm^3^ cell size; Proptek North America, Watsonville, CA). Transplants were grown in a greenhouse before being planted to field beds. Field beds were 20 cm high and 81 cm wide and had been fumigated with a combination of chloropicrin and 1, 3-dichloropropene (Pic-Clor 60 EC, Soil chemical corporation, Hollister, CA, at 300 lbs per treated acre) and covered with black plastic mulch before transplanting. Beds were spaced 152 cm apart, and TRANSPLANTS were planted in a single row within each bed. In-row plant spacing was 46 cm. The tomato plants were staked and tied, and irrigation was applied through drip tape beneath the plastic mulch of each bed. A recommended fertilizer and pesticide program was followed throughout the growing season.

### The effect of large (4.3 and 5.1 Mb) I-3 introgressions on fruit size

Two UF/IFAS parental lines segregating for *I-3*, Fla. 7907B and Fla. 8814, were used for evaluating the effect of the gene on fruit size. These lines contain *I-3* on introgressions approximately 5.1 Mb (Fla. 7907B) and 4.2 Mb (Fla. 8814) in size (Li et al. 2018). Segregation for the locus was discovered in F_12_ plants of Fla. 7907B and in F_4_ plants of Fla. 8814, and these were used for the spring 2015 trial. Seedlings were genotyped with markers linked to *I-3,* and transplants were grouped according to genotype (*i-3/i-3*, *i-3/I-3*, and *I-3/I-3*). Seed was sown on Jan 22, 2015 for the spring 2015 trial and transplanted to the field Feb 27, 2015. A randomized complete block design (RCBD) was used with 3 replications of 8 plant plots. A single plant of each line heterozygous for the *I-3* locus was harvested for seed, and these were used for the next trial. For the spring 2016 trial, seed was sown on Feb 1, 2016 and genotyped and grouped as previously described. Transplants were taken to the field March 21, 2016, and a RCBD was used with 4 replications of 12 plant plots. In both trials, mature fruit were harvested three times and graded using USDA standards. Fruit numbers and weight were recorded for each size category (S, M, L, XL), and average fruit size was calculated. Data were tested for normality of residuals and constant variance prior to data analysis. Data were analyzed by analysis of variance (ANOVA) using PROC GLIMMIX in SAS (version 9.4; SAS Institute, Cary, NC, USA) with a mixed model approach in which blocks were nested within season and both block and season were treated as random effects.

### Reducing the I-3 introgression

**Fig. 1:**
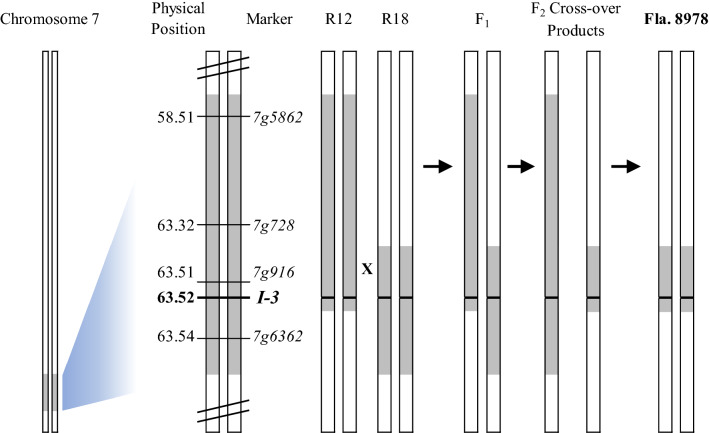
Breeding strategy used to obtain a minimal *I-3* introgression. The original introgression size exceeded 5.0 Mb. Recombinant inbred lines (RILs; derived from R12 and R18 (Li et al. 2018)) resulting from recombination events on either side of *I-3* were intercrossed, and the F_1_ was self-pollinated. Flanking markers 7g728 and 7g6362 were used to screen an F_2_ population and identify the desirable of two possible products of crossing-over which resulted from further recombination within the overlapping homologous region. A plant containing the minimal *I-3* introgression was self-pollinated to obtain a homozygous breeding line, Fla. 8978

An approach similar to that of Lim et al. (2006) was utilized to reduce the *I-3* introgression using RILs R12 and R18 from Li et al. (2018), which had opposing, overlapping portions of the introgression (Fig. 1). R12 resulted from a recombination event between 63.52 Mb (position of *I-3*) and 63.54 Mb, resulting in an introgression that contained *I-3* and the proximal portion of the introgression. R18 resulted from recombination between 63.32 and 63.51 Mb, resulting in an introgression that contained *I-3* and the distil portion of the introgression. Physical positions correspond to the SL4.0 tomato genome assembly (Fernandez-Pozo et al. 2015). R12 and R18 were intercrossed, and the F_1_ was self-pollinated (Fig. 1). Flanking markers were used to screen the F_2_ population and identify the desirable of two possible products of crossing-over which resulted from recombination within the overlapping homologous region. A plant containing the minimal *I-3* introgression was self-pollinated to obtain a homozygous breeding line, Fla. 8978, which was used as the donor for backcrossing the minimal *I-3* introgression into a panel of elite UF/IFAS breeding lines.

To determine the size of the minimal *I-3* introgression, whole genome sequencing of five tomato lines was conducted using Illumina next generation sequencing technology, as described previously (Lee et al. 2018). Fla. 7946(*mI-3*) contained the minimal *I-3* introgression, Fla. 7907B and Fla. 7946 are *Fol3*-resistant breeding lines which carry *I-3* on large introgressions (5.1 Mb and 4.2 Mb, respectively (Li et al. 2018)), and Fla. 8059 and Fla. 8111B are *Fol3-*susceptible breeding lines. For each line, the approach described by Lee et al. (2018) was used to identify SNPs compared with the most recent tomato genome assembly SL4.0 (Fernandez-Pozo et al. 2015).

### Fol3 *disease assays*

Seedling disease assays were conducted to confirm the resistance to *Fol3* conferred by the minimal *I-3* introgression and *I-7*. Both genes were backcrossed into UF/IFAS breeding lines including Fla. 7946, Fla. 8059, and Fla. 8814(*i-3/i-3*). Fla. 8059 and Fla. 8814(*i-3/i-3*) are *Fol3* susceptible, and near isogenic lines (NILs) containing the minimal *I-3* or *I-7* and ‘Tristar’ were compared to susceptibility. NILs were also used to compare the minimal *I-3* introgression to the large (4.2 Mb) introgression using Fla. 7946 and Fla. 8814(*I-3/I-3*) (Li et al. 2018).

Inoculum was prepared by growing the fungus (*Fol3* isolate GEV1400) on potato dextrose agar media at 28 °C for approximately one week, and then removing the fungus from the agar into 100 ml deionized water and adjusting the suspension of fungal conidia. Three concentrations (approximately 10^5^, 10^6^, and 10^7^ spores per ml) were used and conducted as separate disease assays. Seeds were sown in Black Beauty spent coal (Harsco Minerals International, Mechanicsburg, PA, USA). Seedlings were inoculated approximately two weeks after sowing by gently removing them from the coal and dipping the roots into the conidia suspension before transplanting into peat-lite soilless media in 128-well Speedling® trays. Dead plants or those with clear stunting, yellowing, wilting, enlarged stem, and stem collapse were considered susceptible. For plants that did not show clear external symptoms, the root was dissected to determine if vascular browning was present, which is indicative of *Fol* infection. Symptomless, healthy plants were considered resistant.

Disease assays were repeated twice, and data were pooled. Chi-square analyses were used to test for significant differences in the proportions of healthy plants.

### Bacterial spot and fruit size trials of minimal I-3 introgression and I-7

The minimal *I-3* introgression was backcrossed from Fla. 8978 into UF/IFAS breeding lines; Fla. 7907B(*i-3/i-3*), Fla. 7946, Fla. 8059, and Fla. 8814(*i-3/i-3*). Fla. 7946 is an *Fol3*-resistant breeding line containing *I-3* on an approximately 4.2 Mb introgression (Li et al. 2018), and backcross F_2_ populations segregated for this introgression and for the minimal introgression. Molecular marker *7g728* (63.32 Mb) was used to determine which introgression size was present. Fla. 7907B(*i-3/i-3*), Fla. 8814(*i-3/i-3*), and Fla. 8059 are *Fol3* susceptible parents, and backcross F_2_ populations segregated for the minimal *I-3* introgression. To test the effect of *I-7* in a Florida-adapted background, Fla. 8059 served as recurrent parent and ‘Tristar’ was used as the donor of *I-7*. Backcross F_2_ populations segregating for *I-7* were used in field evaluations.

Field trials consisted of inoculation with *X. perforans*, disease scoring, and harvesting and grading of fruit. Inoculum was prepared by growing four strains of *X. perforans* race T4 (GEV904, GEV917, GEV1001, GEV1063) on Difco nutrient agar (Becton–Dickinson and Company, Sparks, MD) for 24–36 h at 28 °C. Bacterial colonies were removed from the agar plates and suspended in 10 mM MgSO4·7 H2O at a concentration of approximately 10^6^ colony forming units per ml with Silwet L-77 (0.025% v/v) (Helena Agri-Enterprises, Collierville, TN). Inoculum was applied to plants approximately 3 weeks after transplanting by misting the foliage with a backpack sprayer. Foliar disease severity was rated using the Horsfall-Barratt scale (1945) where 1 = 0%, 2 = 0–3%, 3 = 3–6%, 4 = 6–12%, 5 = 12–25%, 6 = 25–50%, 7 = 50–75%, 8 = 75–87%, 9 = 87–94%, 10 = 94–97%, 11 = 97–100%, and 12 = 100% diseased tissue. Mature fruit were harvested three times, and fruit were sized according to USDA standards. Fruit numbers and weight were recorded for each size category (S, M, L, XL), and average fruit size was also calculated.

Field trials were conducted from spring 2017 to spring 2019. For each trial, segregating populations were tested with appropriate markers and sorted according to genotype. A Fla. 7946 population segregating for the 4.2 Mb and minimal introgressions was tested across five seasons (spring and fall 2017, spring and fall 2018, and spring 2019). A Fla. 8059 population segregating for the minimal *I-3* introgression was tested across four seasons (fall 2017, spring and fall 2018, and spring 2019). Populations of Fla. 7907B(*i-3/i-3*) and Fla. 8814(*i-3/i-3*) segregating for the minimal introgression were tested across three seasons (spring and fall 2018, spring 2019). Finally, a Fla. 8059 population segregating for *I-7* was tested for two seasons (fall 2018 and spring 2019). Each trial included a RCBD with six blocks of 12-plant plots for all seasons and populations with the exception of the spring 2017 trial with the Fla. 7946 population, which included four blocks of 18-plant plots. Each plot served as one experimental unit.

The effect of *Fol3* resistance (conferred by the 4.2 Mb *I-3* introgression, by the minimal *I-3* introgression, or by *I-7*) was analyzed within each population separately because they were conducted as separate experiments. Horsfall-Barratt ratings were scored on individual plants, and a mean disease severity rating was calculated per plot. The data were tested for normality of the residuals and homogeneity of variance prior to analysis using the Shapiro-Wilks test in SAS (version 9.4, SAS Institute Inc., Cary, NC) with PROC UNIVARIATE. The distribution of the residuals of the scaled data met the assumptions of normality, and data were analyzed by ANOVA with a mixed model approach using PROC GLIMMIX in SAS. For presentation purposes, disease severity ratings were converted to percent disease using midpoints of the ranges described previously. Fruit size data were also checked for normality of residuals and constant variance and analyzed by ANOVA using PROC GLIMMIX in SAS. For the analyses of both traits, blocks were nested within seasons, both block and season were considered random effects, and means were compared using the least squares means statement in SAS with the Tukey’s adjustment (*P* = 0.05).

## Results

### *Large* I-3 *introgression results in a significant decrease in fruit size*

Fla. 7907B and Fla. 8814 are UF/IFAS parental lines which were found to be segregating for the *I-3* locus. These lines contain *I-3* on introgressions of approximately 5.1 Mb (Fla. 7907B) and 4.2 Mb (Fla. 8814), which represent common sizes of *I-3* introgressions found in many public and private breeding lines and commercial hybrids (Li et al. 2018). Segregating populations derived from each of these lines were used to determine the effect of these 4 and 5 Mb *I-3* introgressions on fruit size. Populations were genotyped with markers linked to *I-3*, and plants were grouped according to genotype. Evaluations took place in the spring seasons of 2015 and 2016, and results are presented in Table 1. The *I-3* introgression in both populations had a negative and additive effect on fruit size, with susceptible plants producing the largest fruit, and each copy of *I-3* further reducing fruit size. Fla. 7097B(*i-3*/*i-3*) produced 191 g fruit in the absence of *I-3*, and each *I-3* allele reduced fruit size by an average of 21 g, resulting in a 41 g decrease in fruit size in Fla. 7907B(*I-3*/*I-3*). Similarly, in the Fla. 8814 population, heterozygosity resulted in an intermediate fruit size between homozygous resistant and homozygous susceptibility, with each *I-3* allele contributing to an approximately 20 g reduction in size. There was a significant difference detected in the Fla. 7907B population for yield (kg per plant), for which heterozygous plants had higher yield than plants homozygous for *I-3*. This trend, however, was not observed for the Fla. 8814 population. Fruit number per plant was not significantly different among *I-3* genotypes for either population (Table 1).

**Table 1:** Average fruit size of UF/IFAS breeding lines segregating for large *I-3* introgression

Genotype		Fruit size (g)	Fruit (no./pl)	Yield (kg/pl)
Fla. 7907B	−/−^z^	191 a^y^	25 ns	4.6 ab
	−/+	175 b	29	5.0 a
	+/+	150 c	25	3.7 b
Fla. 8814	−/−	182 a	30 ns	5.3 ns
	−/+	163 b	34	5.5
	+/+	143 c	34	4.7

^z^“−” indicates *S. lycopersicum* (susceptible) allele and “+” indicates *S. pennellii* (resistant) allele at *I-3*

^y^For each genotype, means within a column followed by different letters are significantly different at *P* < 0.05 using Tukey adjusted means comparisons

## I-3 *introgression reduced from 5 Mb to approximately 140 kb*

**Fig. 2:**
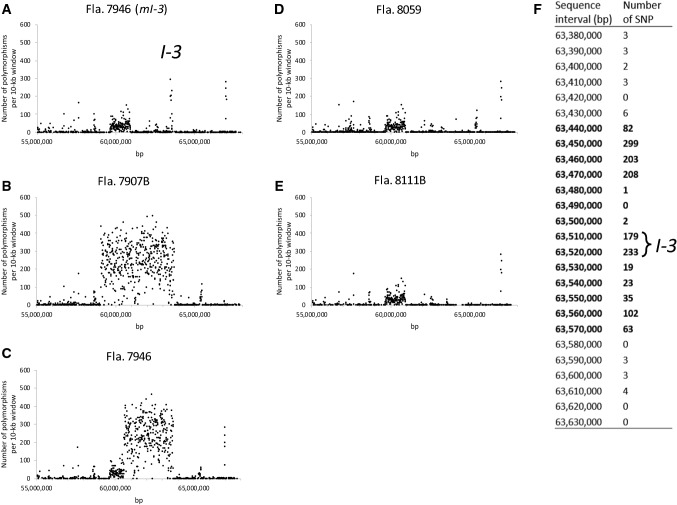
SNP density plot of the *I-3* locus and flanking region. A, B and C show SNP densities for three *I-3* lines (Fla. 7946(*mI-3*), Fla. 7907B, Fla. 7946), and D and E shows SNP densities for two susceptible lines (Fla. 8059 and Fla. 8111B). F shows increased SNP number occurring within an ~ 140 kb region of introgression in Fla. 7946(*mI-3*). Physical positions are based on alignments with the SL4.0 tomato genome assembly (Fernandez-Pozo et al. 2015)

Figure 1 depicts the crossing scheme utilized with RILs containing different portions of the *I-3* introgression (Li et al. 2018) to obtain a reduced *I-3* introgression. R12 contained the proximal portion of the *I-3* introgression (including *I-3*, at 63.52 Mb) and R18 contained *I-3* and the distil portion of the introgression. After intercrossing these and identifying the desirable crossover product in the F_2_, Fla. 8978 was obtained which contained the *I-3* minimal introgression (Fig. 1). Using additional markers, this introgression was determined to be between 70 and 160 kb in size (data not shown). To more precisely determine the size of this introgression, we analyzed WGS-based SNP data from five tomato lines. Given that higher rates of SNPs exist between cultivated and wild tomato, SNP frequency was used to define the introgression boundaries. In Fla. 7946 and Fla. 7907B, the number of SNPs rose sharply to almost 300 per 10 kb window within the respective *S. pennellii* introgressions (Fig. 2b and c), and Fla. 8059 and Fla. 8111B had no regions with such high rates of SNP polymorphism (Fig. 2d and e). In the Fla. 7946 minimal *I-3* introgression NIL, SNP frequency peaked between 63.44 and 63.57 Mb, determining the minimal *I-3* introgression size to be approximately 140 kb (Fig. 2a and f). Based on currently available gene models, there are only 14 annotated *S. pennellii* genes, including *I-3*, in this interval (according to the SL4.0 tomato genome assembly and ITAG 4.1 gene annotations).

## *Minimal *I-3* introgression and *I-7* confer resistance to* Fol3

Seedling disease assays were performed using NILs containing the minimal *I-3* introgression or *I-7*. NILs in the Fla. 7946 and Fla. 8814 backgrounds compared resistance conferred by the minimal and large (4.2 Mb) *I-3* introgressions and demonstrated that the minimal *I-3* introgression gives resistance equal to that of the 4.2 Mb introgression (Table 2). The *I-7* gene was also backcrossed into Fla. 8059, and this NIL and ‘Tristar’ were subjected to *Fol3* disease assays. The *I-7* genotypes gave an intermediate disease response, in that they were significantly better than Fla. 8059, but not as effective as *I-3* (*P* < 0.001) (Table 3). Because resistance conferred by *I-7* was suspected to be affected by inoculum concentration (Scott and Jones 1985; Volin and Jones 1983), assays using 10^7^, 10^6^, and 10^5^ spores/mL were conducted. In all experiments, the proportion of healthy plants of both Fla. 8059(*I-7*) and ‘Tristar’ was significantly greater compared to the *Fol3* susceptible control, Fla. 8059, and significantly less compared to lines with either *I-3* introgression (Table 3). The proportions of healthy plants in the *I-7* lines tested were significantly impacted by the concentration of inoculum, with lower penetrance of resistance at higher concentrations. Fla. 8059(*I-7*) exhibited an intermediate response at 10^6^ spores/mL with a significantly greater percentage of healthy plants compared to 10^7^ (χ^2^ = 45.1237, *P* < 0.001) and a significantly lower percentage than when challenged at 10^5^ spores/mL (χ^2^ = 67.6185, *P* < 0.001). The response of Tristar was similar with percentage of healthy plants at 10^6^ spores/mL intermediate and significantly greater than 10^7^ spores/mL and lower than 10^5^ spores/mL (χ^2^ = 41.1056, *P* < 0.001 and χ^2^ = 63.6469, *P* < 0.001, respectively).Interestingly, Fla. 8059 also demonstrated a low level of resistance at lower inoculum concentrations, with the percentage of healthy plants at 10^6^ spores/mL being greater than that at 10^7^ spores/mL (χ^2^ = 50.2274, *P* < 0.001) and less than that at 10^5^ spores/mL (χ^2^ = 25.2466, *P* < 0.001).

**Table 2:** Disease reaction of plants with the minimal or large (4.2 Mb) *I-3* introgressions challenged with *Fol3*

Breeding line^z^	*I-3* introgression^y^	Total no. plants	Healthy (%)
Fla. 8814	None	119	0
	Large	119	98
	Minimal	118	98
Fla. 7946	Large	126	98
	Minimal	126	100
Horizon	None	128	1
Bonny Best	None	128	0

^Z^The minimal *I-3* introgression was backcrossed into Fla. 8814 which has a large *I-3* introgression; and the resulting near isogenic line (NIL) was compared with Fla. 8814 and with an Fol3 susceptible NIL of Fla. 8814. The minimal *I-3* introgression was also backcrossed into Fla. 7946 which has a large *I-3* introgression; and the resulting NIL was compared with Fla. 7946. Horizon and Bonny Best are *Fol3* susceptible controls

^y^“Large” indicates the NIL homozygous for the large (4.2 Mb) *I-3* introgression, “Minimal” indicates the NIL homozygous for the minimal (140 kb) *I-3* introgression

**Table 3:** Disease reaction of plants with the minimal *I-3* introgression or *I-7* compared with susceptible plants inoculated with *Fol3*

	10^5^^y^		10^6^		10^7^	
Genotype^z^ plants	Total no. of plants	Healthy (%)	Total no. of plants	Healthy (%)	Total no. of plants	Healthy (%)
Fla. 8059	253	56	254	23	251	2
Fla. *8059(mI-3* )	281	99**^x^	272	100**	256	100**
Fla. 8059*(I-7* )	245	86**	242	52**	253	23**
Tristar	229	72**	236	36*	229	10*
Fla. 7946	72	100**	73	99**	64	98**
Horizon	62	24**	64	3*	64	2
Bonny Best	64	9**	64	3*	64	0

^z^Fla. 8059(mI-3) and Fla. 8059(I-7) are near isogenic lines with the minimal *I-3* introgression and *I-7*, respectively. Tristar contains the *I-7* gene and Fla. 7946 contains *I-3* on the large (4.2 Mb) introgression. Fla. 8059, Horizon, and Bonny Best are *Fol3* susceptible

^y^Three separate experiments were conducted with spore suspensions of 10^5^, 10^6^, and 10^7^ spores per mL. Each experiment was repeated twice and data were pooled for analysis

^x^Asterisks denote percentage of healthy plants that are significantly different from Fla. 8059 (*Fol3* susceptible) as determined by chi-square analyses at < 0.001 (**) and < 0.01 (*)

### Minimal I-3 introgression results in less bacterial spot and larger fruit size than the 4 Mb I-3 introgression

To compare effects of the different *I-3* introgressions, Fla. 8978 was crossed to Fla. 7946, which contained *I-3* on an approximately 4.2 Mb introgression (Li et al. 2018). Molecular marker *7g728* was used to distinguish between the two introgression sizes, and segregating backcross F_2_ populations were used for evaluations, ensuring the comparisons made between introgressions were done in a single background, reducing variation. Results are presented in Table 4. Data from the five field seasons demonstrates significant differences between the haplotypes. Homozygosity for the 4.2 Mb *I-3* introgression resulted in significantly higher disease severity compared to heterozygosity or homozygosity for the minimal introgression, and plants homozygous for the minimal *I-3* introgression had the lowest disease severity, though not significantly different from the heterozygous group. Data for average fruit size reflected a similar trend. The average fruit size of plants homozygous for the minimal *I-3* introgression was 173 g, an increase of 27 g compared to the plants homozygous for the 4.2 Mb introgression. The 4.2 Mb introgression was again observed to have a dosage effect on fruit size, where heterozygosity intermediate in fruit size, though not significantly different from homozygous for the minimal introgressions. Significant differences were not observed for total yield or fruit number among the haplotypes.

**Table 4:** Bacterial spot disease severity and average fruit size of Fla. 7946 populations segregating for the 4 Mb and minimal *I-3* introgressions

	Disease	Fruit Size	Fruit	Yield
Genotype	(%)^y^	(g)	(no./pl)	(kg/pl)
Large/large^z^	55 a	146 b	48 ns	6.9 ns
Minimal/large	53 b	165 a	44	7.3
Minimal/minimal	51 b	173 a	41	6.9

^z^Large" indicates the *I-3* allele on the 4.2 Mb introgression and "Minimal" indicates *I-3* on the minimal (140 kb) introgression

^y^Percent diseased tissue as converted from Horsfall-Barratt ratings

^x^Means followed by different letters are significantly different at *P* < 0.05 using Tukey adjusted means comparisons

### No effect of minimal I-3 introgression or I-7 on bacterial spot or fruit size compared to susceptibility

While it was encouraging that reducing the size of the *I-3* introgression resulted in less bacterial spot and increased fruit size compared to the 4.2 Mb introgression, it was critical to test whether there may still be effects of the minimal *I-3* introgression relative to susceptibility. Furthermore, the *I-7* gene was previously available in very limited material, none that was adapted to Florida’s climate, and no information was available about potential association with detrimental effects. To test the minimal *I-3* introgression for association with negative traits, backcross populations were developed using *Fol3* susceptible UF/IFAS breeding lines (Fla. 7907B(*i-3/i-3*), Fla. 8814(*i-3/i-3*) and Fla. 8059) as recurrent parents, and *I-7* was backcrossed into Fla. 8059. F_2_ populations were obtained for each line and grouped by genotype with the appropriate molecular markers, then evaluated for effects on bacterial spot and fruit size in field trials. Results are presented in Table 5. In each background, the minimal *I-3* introgression had no effect on bacterial spot sensitivity or on fruit size within any line. There was also no difference detected among genotypes of Fla. 8059 with *I-7* for either disease severity or fruit size.

**Table 5:** Bacterial spot and average fruit size in near isogenic backgrounds segregating for either the minimal *I-3* introgression or *I-7* compared to susceptibility

			Disease	Fruit size
			(%)^y^	(g)
Minimal *I-3* introgression	Fla. 7907	−/− ^z^	30ns^x^	181 ns
		−/+	28	181
		+/+	30	189
	Fla. 8059	−/−	31 ns	149 ns
		−/+	31	145
		+/+	31	146
	Fla. 8814	−/−	38 ns	166 ns
		−/+	39	163
		+/+	39	166
*I-7*	Fla. 8059	−/−	19 ns	148 ns
		−/+	19	146
		+/+	19	151

^z^“−” indicates *S. lycopersicum* (susceptible) allele and “+” indicates *S. pennellii* (resistant) allele at *I-3*

^y^Percent diseased tissue as converted from Horsfall-Barratt ratings

^x^"ns" indicates means for genotypes within each near isogenic background are not significantly different by * F* test at * P* < 0.05

## Discussion

Fusarium wilt race 3 is capable of causing devastating crop loss in tomato growing regions around the world, and control through chemical or cultural means is inadequate. Host resistance via the *I-3* gene is the best management strategy, but breeders have struggled to develop superior *Fol3*-resistant cultivars, partly due to the association between *I-3* and negative traits. Bacterial spot is another major disease of tomato that causes significant damage and crop loss in humid tomato production regions. This disease is also very difficult to control with chemical or cultural practices, and there are no commercially available resistant cultivars. In production areas such as Florida where both bacterial spot and *Fol3* are a concern for growers, cultivars are needed with both bacterial spot tolerance and *Fol3* resistance. The association between *I-3* resistance and increased sensitivity to bacterial spot has challenged tomato breeders since the gene’s introduction more than 30 years ago. Hutton et al. (2014) found *I-3* to contribute as much as 20% increase infection to bacterial spot. However, it was recently suggested this association is likely due to negative loci linked to *I-3* rather than an effect of pleiotropy, and that linkage with these alleles could be broken (Li et al. 2018). In the case of fruit size, Scott (1999) showed a negative effect of *I-3* on fruit size in homozygous-resistant plants compared to heterozygosity. However, it was unknown whether this effect is due to pleiotropy or linkage drag, or how resistance compares with susceptibility. *I-7* is an additional *Fol3* resistance gene available (Gonzalez-Cendales et al. 2016), although it has been less widely utilized, and it is unknown whether *I-7* is associated with similar negative traits as *I-3*. Whereas it would be beneficial to deploy both *Fol3* resistance genes together, the usefulness of *I-7* depends on the gene being free from association with negative traits. Our objectives were to determine the extent to which *I-3* affects fruits size, reduce the size of the *I-3* introgression, and evaluate whether linkage with these negative traits could be broken. We also characterized *I-7* for effects on these traits, to determine whether any negative associations existed.

Fruit size is a particularly important fruit-quality trait for fresh-market tomato growers due to the needs of stakeholders, notably food service industries and grocers who generally pay higher prices for larger sizes. The association between *I-3* and reduced fruit size has been noted by breeders for many years (Scott 2004, 1999). However, comparisons were only made between plants homozygous and heterozygous for resistance by Scott (1999). Since the time of that study, breeding efforts have continued, and the effect of the *I-3* introgression in more modern germplasm is unknown. We developed populations with elite parental UF/IFAS breeding lines Fla. 7907B and Fla. 8814, which segregated for *I-3* on introgressions of approximately 5 and 4.2 Mb, respectively (Li et al. 2018). Homozygosity for either the 4 or 5 Mb *I-3* introgressions resulted in a decrease of fruit size by approximately 21% compared to susceptibility. The effect was also found to be additive, where the heterozygous state resulted in an intermediate fruit size between plants homozygous resistant or susceptible. This is consistent with the findings of Scott (1999) and demonstrates that the challenges with fruit size persist in modern *I-3* breeding materials and are not exclusive to early generation *I-3* germplasm. Although fruit size is commonly inversely related to yield, previous reports did not observe significant effects of *I-3* on yield. We detected significant variation for yield in the Fla. 7907B population (Table 1), but given the lack of an effect in the Fla. 8814 population combined with the lack of an effect on fruit number for either population, we deem this association weak, at best. Furthermore, this association would have little utility for yield improvement since the advantage of heterozygosity was only significant relative to the homozygous resistant genotype.

We hypothesized that the association between *I-3* and the unfavorable alleles contributing to both increased bacterial spot sensitivity and reduced fruit size could be eliminated by reducing the size of the *I-3* introgression. Utilizing a strategy similar to that described by Lim et al. (2006), *I-3* RILs with opposing segments were used to reduce the introgression from over 5 Mb to a minimal *I-3* introgression that is approximately 140 kb in length (Fig. 1; Fig. 2). Previously, Li et al. (2018) surveyed current *Fol3*-resistant breeding lines and commercial hybrids to determine the sizes of the *I-3* introgressions they contained and found the introgressions to range from greater than 5 Mb to between 1.0 and 2.7 Mb. Our work to produce the minimal *I-3* introgression provides tomato breeders with *I-3* on the smallest introgression currently known.

Reducing the *I-3* introgression did not affect *Fol3* resistance as demonstrated by disease screens. Lines containing *I-7* were also subjected to *Fol3* disease assays, and inoculum concentration had a significant effect on resistance. Effects of inoculum concentration on resistant materials from Australia, where *I-7* was introgressed and ‘Tristar’ developed, have been reported previously, where lower inoculum rates were required to avoid killing most plants (Scott and Jones, 1985; Volin and Jones, 1983). Our *Fol3* disease screens also demonstrated that although both ‘Tristar’ and Fla. 8059(*I-7*) had significantly greater percentages of healthy plants compared to *Fol3* susceptible controls, these lines had significantly more disease compared to those containing *I-3*, and resistance was reduced by higher inoculum concentrations. This characteristic was not reported by Gonzalez-Cendales et al. (2016) when describing race 3 resistance conferred by *I-7* in seedling disease screens, and the discrepancy could be attributed to differences in inoculation and screening methods. In the present study, plants were only scored as resistant if they were free of vascular browning in both the stems and roots, and this stringent definition could potentially exclude plants with slight vascular browning but an otherwise acceptable level of field resistance*.* Alternatively, the differences could be indicative of variation among *Fol3* isolates where those obtained from Florida may have greater virulence than isolates used by Gonzalez-Cendales et al. (2016). Either scenario warrants further investigation. The low level of resistance observed in Fla. 8059 relative to other susceptible controls suggests the presence of a minor resistance allele(s) in this line, which may account for the greater degree of resistance in the Fla. 8059 *I-7* NIL relative to ‘Tristar.’

To determine what effect reducing the *I-3* introgression had on bacterial spot sensitivity and fruit size, the minimal introgression was incorporated into UF/IFAS breeding lines via backcrossing, and segregating populations were evaluated in field trials. One of these breeding lines, Fla. 7946, contains the *I-3* gene on an approximately 4.2 Mb introgression, and this population was utilized to directly compare the two introgression sizes. We found that homozygosity for the 4.2 Mb *I-3* introgression contributed an approximately 15% increase in disease severity compared to homozygosity for the minimal introgression. Li et al. (2018) previously found *I-3* to contribute additively to bacterial spot sensitivity, where heterozygosity resulted in an intermediate disease response compared to either homozygous state, and our results were consistent with this finding. We also found that fruit size was significantly impacted by the size of the *I-3* introgression, where homozygosity for the minimal introgression resulted in a gain of nearly 30 g in average fruit size compared with the 4.2 Mb introgression. These results support that the association between *I-3* and both increased bacterial spot sensitivity and reduced fruit size is due to linkage drag, and that the minimal *I-3* introgression no longer contains the unfavorable alleles contributing to these traits. Whereas Li et al. (2018) showed that bacterial spot sensitivity was associated with the proximal portion of the *I-3* introgression, above 63.41 Mb, there was no indication whether the association with fruit size was due to a similar linkage drag effect or pleiotropy. Wild tomato species are well known to be associated with small fruit, which often weigh no more than a few grams, and several major quantitative trait loci (QTLs) have been identified in *S. pennellii* that contribute to small fruit size with additive effects (Tanksley 2004; Eshed and Zamir 1995). Although there are no reports of fruit size QTLs located near *I-3*, it is probable that the effect on fruit size is due to an unfavorable *S. pennellii* QTL near *I-3*, and reducing the introgression size has resulted in breaking linkage with this trait as well.

To further support our hypothesis, three additional populations were generated with Fla. 8059, Fla. 7907B(*i-3/i-3*), and Fla. 8814(*i-3/i-3*) to determine whether the minimal *I-3* introgression has any negative effect in *Fol3* susceptible material. We found no effect of the minimal introgression compared to susceptibility for either bacterial spot disease severity or average fruit size. Taken together, these results demonstrate that the minimal *I-3* introgression is free from association with these two detrimental traits, and germplasm containing this minimal introgression is available. The *I-7* gene was also evaluated for potential effects on bacterial spot and fruit size in a Fla. 8059 backcross population, but no effect was associated between *I-7* and either of these traits.

The negative associations with *I-3* may help to explain why *Fol3* resistance has been slow to become prevalent among cultivars, in contrast with *I* and *I-2* which are commonly deployed in cultivars around the world. Our results may facilitate the more comprehensive advancement of *Fol3* resistance into additional commercial backgrounds such as processing tomato cultivars. California leads the USA in processing tomato production (NASS 2020), and although the state has increased incidence of *Fol3*, few processing tomato cultivars utilize the *I-3* gene. It is possible that the slow rate of deployment of *I-3* in such markets is due to breeding challenges such as we have described, and the minimal *I-3* introgression may therefore be of particular benefit to incorporate *Fol3* resistance into this important industry.

With the availability of two genes free of association with these detrimental traits, tomato breeders may now deploy the minimal *I-3* and *I-7* genes together to extend the durability of *Fol3* resistance. Single, dominant resistance genes are predominantly used in tomato breeding when available, rather than quantitatively mediated resistance, largely due to the ease of breeding with major genes, especially for development of hybrid cultivars. However, this type of resistance may be overcome by pathogen mutation. In the case of *Fol*, mutations in effector proteins, encoded by *Secretion in xylem* (*Six*) genes (Takken and Rep 2010), has led to the breakdown of some tomato resistance genes, thus multiple races of the pathogen. After the introduction of *I* in the 1940′s, *Fol2* strains were isolated from plants in fewer than 10 years, and *I-2* was overcome approximately 20 years after deployment (Takken and Rep 2010). Although *I-3* resistance has proven more stable since its introduction more than 30 years ago, its durability may be improved by combination with *I-7*. Pyramiding resistance genes is an established approach used effectively in many crops to create more durable resistance by combining multiple resistance genes into a single genotype, especially with genes representing different modes of action between the resistance genes and their associated *Avr* effectors (Douglas and Halpin 2010; Zhu et al. 2012). Given that *I-3* encodes an S-receptor-like kinase, *I-7* encodes a leucine-rich repeat receptor-like protein, and the resistance conferred by these genes is likely dependent on different pathogen effectors, pyramiding *I-3* and *I-7* may improve the durability of these genes (Gonzalez‐Cendales et al. 2016). Moreover, the efficacy of *I-3* may be further extended by continuing to deploy this gene in combination with the *I* gene. Although it has been reported that the *I-3* introgression also confers resistance to *Fol1* and *Fol2*, Houterman et al. (2008) reported that the *Avr1* (*SIX4*) gene carried by race 1 suppresses *I-3* mediated resistance, and thus *I-3* would no longer provide effective resistance if there were a resurgence of strains that carry *Avr1*. Similarly, the deployment of *I-3* in combination with *I-7* would help to prevent such a resurgence, since *I-7* was also reported to confer resistance to *Fol1* and *Fol2* and does not appear to be suppressed by *Avr1* (Gonzalez-Cendales et al. 2016).

The literature is not clear whether *I-3*-mediated resistance to *Fol1* and *Fol2* is due to the *I-3* gene itself or to other tightly linked genes. Sarfatti et al. (1991) and Scott et al. (2004) proposed a putative *Fol1* resistance gene, *I1*, contained within the *I-3* introgression, and Scott et al. (2004) also found evidence of a possible *Fol2* resistance gene tightly linked with *I-3*. In contrast, Do et al. (2016) reported that the *I-3* gene itself confers resistance to races 1 and 2, and they found no evidence for additional resistance genes linked to *I-3*. If additional resistance genes linked to *I-3* exist, reducing the *I-3* introgression to resolve linkage drag effects may have had the unintentional consequence of disassociating such loci from *I-3*. Therefore, it will be important to characterize the minimal *I-3* introgression for resistance to *Fol1* and *Fol2* to better understand the situation and to effectively deploy improved *Fol* resistance, and this material is currently under development.

## Conclusion

Breeders have struggled for more than 30 years to develop *Fol3*-resistant tomato cultivars that meet the high standards required by tomato growers partly due to the association between *I-3* and increased bacterial spot sensitivity and reduced fruit size. By reducing the *I-3* introgression from over 5 Mb to 140 kb, germplasm with resistance free of these negative effects is now available. Because the *I-7* gene was not found to have an effect on these two traits, both resistance genes can be combined in order to take advantage of the different ways these genes interact with the pathogen and reducing the likelihood of a simple mutation in the pathogen resulting in the resistance being overcome. This work provides tomato breeders with the material and information needed to develop superior tomato hybrids to develop a more economically sustainable solution to Fusarium wilt for tomato growers throughout the world.

## Data Availability

The datasets generated during and/or analyzed during the current study are available from the corresponding author on reasonable request.

## References

[CR1] Alexander LJ (1959). Progress report of national screening committee for disease resistance in the tomato for 1954–1957. Plant Dis Rptr.

[CR2] Bohn GW, Tucker CM (1939). Immunity to Fusarium wilt in the tomato. Science.

[CR3] Catanzariti AM, Lim GT, Jones DA (2015). The tomato *I-3* gene: a novel gene for resistance to Fusarium wilt disease. New Phytol.

[CR4] Do TTH, Catanzariti A-M, Lim GTT, Jones DA (2016) Evidence against the existence of genes for resistance to *Fusarium oxysporum* f. sp. *lycopersici* races 1 and 2 on *Solanum pennellii* chromosome 7 additional to *I-3*. In: V International Symposium on Tomato Diseases: Perspectives and Future Directions in Tomato Protection 1207, pp 19-26

[CR5] Douglas E, Halpin C, Jain SM, Brar DS (2010). Gene stacking. Molecular techniques in crop improvement.

[CR6] Eshed Y, Zamir D (1995). An introgression line population of *Lycopersicon pennellii* in the cultivated tomato enables the identification and fine mapping of yield-associated QTL. Genetics.

[CR7] Fernandez-Pozo N, Menda N, Edwards JD, Saha S, Tecle IY, Strickler SR, Bombarely A, Fisher-York T, Pujar A, Foerster H, Yan A, Mueller LA (2015). The Sol Genomics Network (SGN) - From genotype to phenotype to breeding. Nucl Acids Res.

[CR8] Gonzalez-Cendales Y, Catanzariti AM, Baker B, McGrath DJ, Jones DA (2016). Identification of *I-7* expands the repertoire of genes for resistance to Fusarium wilt in tomato to three resistance gene classes. Mol Plant Pathol.

[CR9] Hutton SF, Scott JW, Vallad GE (2014). Association of the Fusarium wilt race 3 resistance gene, *I-3*, on chromosome 7 with increased susceptibility to bacterial spot race T4 in tomato. J Am Soc Hort Sci.

[CR10] Katan J (1971). Symptomless carriers of the tomato Fusarium wilt pathogen. Phytopathology.

[CR11] Lee TG, Shekasteband R, Hutton SF (2018). Molecular markers to select for the *j-2*–mediated jointless pedicel in tomato. HortScience.

[CR12] Li J, Chitwood J, Menda N, Mueller L, Hutton SF (2018). Linkage between the *I-3* gene for resistance to Fusarium wilt race 3 and increased sensitivity to bacterial spot in tomato. Theor Appl Genet.

[CR13] Lim G, Wang G-P, Hemming M, Basuki S, McGrath D, Carroll BJ, Jones D (2006). Mapping the *I-3* gene for resistance to Fusarium wilt in tomato: application of an *I-3* marker in tomato improvement and progress towards the cloning of *I-3*. J Australas Plant Pathol.

[CR14] Lim G, Wang GP, Hemming M, McGrath D, Jones D (2008). High resolution genetic and physical mapping of the *I-3* region of tomato chromosome 7 reveals almost continuous microsynteny with grape chromosome 12 but interspersed microsynteny with duplications on *Arabidopsis* chromosomes 1, 2 and 3. Theor Appl Genet.

[CR15] McGrath D, Gillespie D, Vawdrey L (1987). Inheritance of resistance to Fusarium oxysporum f. sp. lycopersici races 2 and 3 in Lycopersicon pennellii. Aust J Agr Res.

[CR17] Noling J, Becker J (1994). The challenge of research and extension to define and implement alternatives to methyl bromide. J Nematol.

[CR18] Ragsdale N, Wheeler W, Kuhr RJ, Roe RM (1995). Methyl bromide: risks, benefits and current status in pest control. Reviews in pesticide toxicology.

[CR19] Sarfatti M, Abu-Abied M, Katan J, Zamir D (1991). RFLP mapping of I1, a new locus in tomato conferring resistance against Fusarium oxysporum f. sp. lycopersici race 1. Theor Appl Genet.

[CR20] Scott J (1999). Tomato plants heterozygous for fusarium wilt race 3 resistance develop larger fruit than homozygous resistant plants. Proc Fla State Hort Soc.

[CR21] Scott J, Agrama H, Jones J (2004). RFLP-based analysis of recombination among resistance genes to Fusarium wilt races 1, 2, and 3 in tomato. J Am Soc Hort Sci.

[CR22] Scott JW, Jones J (1985) An update on Fusarium wilt race 3 resistance. In: Proc Tomato Breeders Roundtable, 1985, p 13

[CR23] Scott J, Jones J (1989). Monogenic resistance in tomato to Fusarium oxysporum f. sp. lycopersici race 3. Euphytica.

[CR24] Scott J (2004). Fla. 7946 tomato breeding line resistant to *Fusarium oxysporum* f. sp. *lycopersici* races 1, 2, and 3. HortScience.

[CR25] Takken F, Rep M (2010). The arms race between tomato and *Fusarium oxysporum*. Mol Plant Pathol.

[CR26] Tanksley SD (2004). The genetic, developmental, and molecular bases of fruit size and shape variation in tomato. Plant Cell.

[CR27] Volin RB, Jones JP (1983) Progress in developing resistance to Fusarium race 3 in Florida. In: Proc 4th Tomato Quality Workshop, p 105

[CR28] Zhu S, Li Y, Vossen JH, Visser RG, Jacobsen E (2012). Functional stacking of three resistance genes against *Phytophthora infestans* in potato. Transgenic Res.

